# Draft Genome Sequences of Eight Crimean-Congo Hemorrhagic Fever Virus Strains

**DOI:** 10.1128/genomeA.00240-17

**Published:** 2017-06-08

**Authors:** J. W. Koehler, K. L. Delp, B. J. Kearney, T. A. Conrad, R. J. Schoepp, A. R. Garrison, L. A. Altamura, C. A. Rossi, T. D. Minogue

**Affiliations:** a Diagnostic Systems Division, United States Army Medical Research Institute of Infectious Diseases, Fort Detrick, Maryland, USA; b Virology Division, United States Army Medical Research Institute of Infectious Diseases, Fort Detrick, Maryland, USA

## Abstract

Crimean-Congo hemorrhagic fever virus (CCHFV) is a geographically widespread RNA virus with a high degree of genomic diversity that complicates sequence-based diagnostics. Here, we sequenced eight CCHFV strains for improved assay design and deposition into FDA-ARGOS, the FDA’s pathogen database for development and verification of next generation sequencing assays.

## GENOME ANNOUNCEMENT

Crimean-Congo hemorrhagic fever virus (CCHFV) is a geographically widespread (1–6) and genetically diverse virus (7–9). Account for this genomic diversity is critical for efficacious diagnostic assay design, as highlighted by Atkinson and colleagues (10). Here, we sequenced each genome segment of multiple CCHFV strains prepared by the Unified Culture Collection (UCC) to improve our CCHFV assay and include in FDA-ARGOS.

Total nucleic acids were acquired from the UCC for eight CCHFV strains including IbAr 10200 (UCC# R4401), DAK8194 (UCC# R4416), SPU 128/81 (UCC# R4417), SPU 115/87 (UCC# R4448), UG 3010 (UCC# R4432), JD-206 (UCC# R4413), HY-13 (UCC# R4459), and Drosdov (UCC# R4405). Each segment was amplified using a previously published protocol (7) with primers modified for Nextera-based sequencing. Amplicon for each genome segment was gel-extracted, processed with the Nextera XT kit (Illumina, San Diego, CA), and sequenced on the MiSeq sequencer (Illumina).

Sequencing reads were analyzed using CLC Genomics Workbench (Qiagen, Valencia, CA). Reads were trimmed for quality and to remove the internal L amplification primer sequences, *de novo* assembled, and BLAST analyzed to identify the closest matching CCHFV sequence. Total reads were mapped again against the virus-specific contigs to generate a final consensus sequence for each genome segment. For JD-206, The L2 segment amplified poorly, resulting in an incomplete assembly. This segment was re-amplified and sequenced using a sequence-optimized L2-F primer (5′-GGAAGAGTTATACAACATAAGGC) modified for Nextera sequencing. The 5′ end of the M segment of JD-206 did not fully assemble, and Sanger sequencing data using the primer CCHF JD-206 MR (5′-TTCCTCCATTGTGAGATGAAGC) was used to complete the assembly.

All segments had at least 100× coverage across the genome. Segments for IbAr 10200 (M segment), Drosdov (M segment), SPU128/81 (M and S segments), UG3010 (L segment), and HY-13 (S segment) had multiple nucleotide variants resulting in amino acid changes and/or in-frame deletions. Sequencing of SPU 128/81 (L segment) and HY-13 (M segment) extended and completed the sequences already in GenBank. Sequences for SPU 115/87 (all segments), the L segment for HY-13, and the L and M segments of JD-206 have not been deposited into GenBank. Sequencing reads for all strains were deposited with NCBI Sequence Read Archive (SRA), and consensus sequences were deposited into FDA-ARGOS as the assembly qualities met database requirements.

Overall, we generated 24 separate CCHFV genome segments from eight different strains. Six new sequences having nonsynonymous variants or in-frame deletions were generated for genome segments already within GenBank. Two segments in GenBank were extended to completion, and five novel segment sequences were completed.

### Accession number(s).

Consensus sequences for each segment were submitted to DDBJ/EMBL/GenBank database under accession no. KY484034, KY484035, KY484036, KY484025, KY484026, KY484027, KY484043, KY484045, KY484044, KY484041, KY484042, KY484040, KY484046, KY484047, KY484048, KY484039, KY484038, KY484037, KY484033, KY484032, KY484031, KY484030, KY484029, and KY484028.
